# Fast visual adaptation to dim light in a cavity-nesting bird

**DOI:** 10.1098/rspb.2023.0596

**Published:** 2023-05-10

**Authors:** Sandra Chaib, Olle Lind, Almut Kelber

**Affiliations:** Lund Vision Group, Department of Biology, Lund University, 223 62 Lund, Sweden

**Keywords:** cavity-nesting birds, budgerigar, vision, dark-adaptation, contrast vision, visual adaptation

## Abstract

Many birds move fast into dark nest cavities forcing the visual system to adapt to low light intensities. Their visual system takes between 15 and 60 min for complete dark adaptation, but little is known about the visual performance of birds during the first seconds in low light intensities. In a forced two-choice behavioural experiment we studied how well budgerigars can discriminate stimuli of different luminance directly after entering a darker environment. The birds made their choices within about 1 s and did not wait to adapt their visual system to the low light intensities. When moving from a bright facility into an environment with 0.5 log unit lower illuminance, the budgerigars detected targets with a luminance of 0.825 cd m^−2^ on a black background. When moving into an environment with 1.7 or 3.5 log units lower illuminance, they detected targets with luminances between 0.106 and 0.136 cd m^−2^. In tests with two simultaneously displayed targets, the birds discriminated similar luminance differences between the targets (Weber fraction of 0.41–0.54) in all light levels. Our results support the notion that partial adaptation of bird eyes to the lower illumination occurring within 1 s allows them to safely detect and feed their chicks.

## 1. Introduction

Birds use vision in a wide range of different light regimes, from bright daylight (approx. 10^5^ lux) to dim starlight under the canopy of trees (approx. 10^−3^ lux [1]). Although some species spend their awake time in dim light conditions, most birds are active in bright daylight, where their visual system allows them to scan the environment rapidly and in great detail [2]. Still, even nocturnal birds can use vision in daylight, and diurnal birds can to some extent see in dim light, which is important, as light levels in natural habitats are highly dynamic. During a day, skylight levels change by a factor of 1000, and a bird flying from an open field into the woods can experience a light intensity decrease by a factor of 100 or more [1]. Thus, bird eyes need continuous adaptation to match the present light conditions.

Vertebrate eyes employ several strategies to adapt to changing light conditions. One strategy is the pupillary light response which controls the amount of light reaching the retina [3]. In most land-living vertebrates, pupil dilation and constriction work within seconds [2–4]. Even though its dynamic range varies between different species, the pupillary light response accounts for a small amount of adaptation only (in budgerigars (*Melopsittacus undulatus*), for instance, changing the retinal illumination by less than a factor of 2 [4]), and its primary purpose has been attributed to other functions such as preventing a decrease in visual acuity in bright light owing to optical aberrations [2–4].

A second adaptation to vision in a broad range of light intensities is the ‘duplex retina’ of vertebrates, with two sets of photoreceptors, rods—active in dim (scotopic) light conditions, and cones—active during bright (photopic) light conditions [5,6]. At intermediate (mesopic) light levels both cones and rods contribute to vision. Birds have an even more complex retina; in addition to rods and cones they have double cones, which can operate under somewhat lower light conditions than single cones [7].

Although the different receptor types allow the visual system to work in a wide intensity range, additional retinal mechanisms, at different light levels and with different time courses are involved in luminance adaptation [8–11].

Prolonged exposure to bright light ‘bleaches’ a part of the photoreceptor pigment which has to be regenerated before it can absorb photons again [12,13]. The recovery of full sensitivity, known as dark-adaptation, can take more than 40 min in humans [13,14] and has a similar time-course in birds [15–17]. Cone recovery is faster and occurs within just a few minutes [13].

In addition, fast mechanisms allow the vertebrate retina to cope with the smaller luminance changes associated with the change of gaze within a visual scene. Response gain is adjusted to the background luminance, enhancing the visual signal at dim backgrounds and preventing saturation in bright light [18]. These mechanisms act, within less than a second, at photoreceptor level and later stages in the retinal pathway [8,18–20].

As a result of luminance adaptation, the visual system can extract detailed information and keep contrast sensitivity largely constant in a wide range of light intensities [9]. The sensitivity of the adapted eye to luminance differences is proportional to the average light intensity, a relation referred to as Weber's law (Δ*I*/*I*_B_ = *k* [9,10]). This linear relationship between background luminance and contrast sensitivity only breaks down at very high light levels owing to saturation of the photoreceptors and at low light levels owing to receptor noise [9].

The visual sensitivity and spatial resolution of birds have been studied after adaption to various light levels, and full dark adaptation has a similar time-course in birds as in humans [14,15]. By contrast, very little is known about the visual capacities of birds after fast changes of luminance. When provisioning their brood, cavity nesting birds may move frequently back and forth between sunlit foraging grounds and the tree cavities or nest-boxes, where they may face light intensities 1000-fold dimmer than outside [21,22]. Visits to the nest often take only a few seconds, probably too short for dark adaptation by slow mechanisms such as photopigment regeneration [15].

Several studies indicate that cavity nesting birds use visual cues when feeding their chicks: after experimental manipulation of the colour of the gape flanges of cavity-breeding passerine nestlings, brighter and more conspicuously coloured individuals gain more weight than their duller siblings [23–25]. Lower luminance contrast between chicks and their background or lower illumination in the nest make food transfer between parent and nestlings more difficult [26,27]. Breeding great tits (*Parus major*) choose brighter nest-boxes over darker ones [28], and in darker nest-boxes, both great tits and marsh tits (*Poecile palustris*) build nest cups closer to the entrance, probably to compensate for the dim illumination [29,30]. Some cavity nesting passerines also use vision to discriminate their eggs from the eggs of brood parasites and assess the fitness of the female (i.e. [31,32]).

Here we use the budgerigar, a species that nests in tree holes in the interior of Australia [33] as a model species to study luminance discrimination of birds that move fast from a bright to a dark environment. Pupil dynamics [4], contrast sensitivity [34–37], spatial acuity [34,36–39] and colour vision [7,34,40–42] of the species have previously been investigated under different light levels. We performed two behavioural experiments to investigate (i) the detection threshold for bright targets on dark background, and (ii) the discrimination threshold for two targets of different luminance.

## 2. Methods

### (a) Animals

Two female and two male budgerigars participated in the experiment. The budgerigars were held in the animal facility at the Department of Biology at Lund University and fed a parakeet seed mix as well as fresh fruit and vegetables. On days of training and testing (3–5 days per week), the seed mix was restricted and only used as a reward. However, the birds had always access to fresh fruit and vegetables in the housing cage. All experiments followed Swedish legislation, under the permit dnr. 5.8.18-17189/2018 granted by the responsible authority (Malmö – Lunds djurförsöksetiska nämnd).

### (b) Experimental set-up

**Figure 1.**
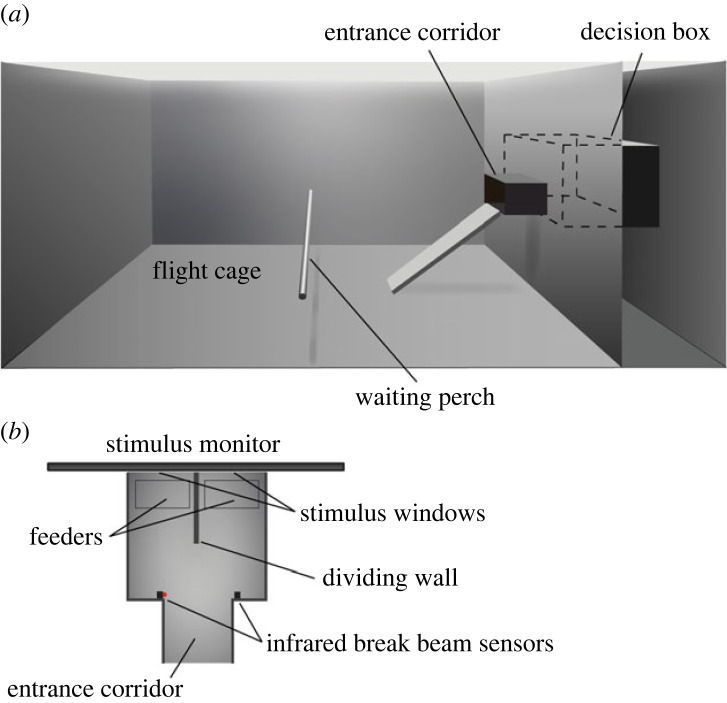
Experimental set-up. (*a*) Side view of the setup. (*b*) The decision box viewed from above.

The experimental set-up comprised two compartments, the large flight cage, and the smaller decision box (figure 1*a*). The flight cage was a net cage, 133 cm long, 65 cm high and 84 cm wide, with a grey plastic floor. A waiting perch was positioned halfway between and parallel to the short walls, and 20 cm above the floor, for the experimental bird. A camera (GoPro Hero, GoPro Inc., San Mateo, CA 94402, USA) on one short wall of the flight cage allowed us to monitor the behaviour of the bird without being seen. The opposite short wall was made of wood and separated the flight cage from the decision box. A bird reached the decision box by flying or by climbing a wooden ramp and entering a corridor (10 cm high and wide and 12 cm long), which protruded into the flight cage from an opening in the wooden wall 25 cm above the floor. This corridor prohibited light from the flight cage from entering the decision box through the opening.

The decision box had solid side walls and was 22 cm long, 20 cm wide and 36 cm high. The wall opposite the entrance was open to a monitor that displayed visual stimuli in two stimulus windows, one to the right and one to the left (figure 1*b*). Decision distance was controlled by a divider that protruded orthogonally from between the stimulus windows 12 cm into the box. A bird entering the decision chamber would view two different stimuli, one on the right and one on the left side of the wall and make a choice by entering the compartment on either side. The floor beneath each stimulus window had a sliding door which was controlled manually by the experimenter from the outside and could be opened to provide a reward of seeds.

Four white LEDs (LZC-00NW40, LED Engin Inc., San Jose, USA) and two fluorescent tubes (Biolux L18W/965; Osram, flicker frequency 9 500 Hz) illuminated the decision box and the flight cage indirectly from above by reflection from wrinkled aluminium foil to ensure an even illumination. This provided an illuminance of 1400 lux at the centre of the waiting perch (measured with a Hagner Luxmeter, Hagner AB, Solna, Sweden, pointing upwards). The illuminance in the smaller decision box was 469 lux (measured the same way as above, with the luxmeter placed between the entrance and the end of the dividing wall, 5 cm above the floor) at the brightest illuminance condition (light level 1). By placing neutral density filters (Lee filters, Andover, Hampshire, UK) on top of the transparent plastic roof of the decision box the light level was dimmed to obtain three additional illuminance conditions: 28 lux (light level 2), 1.83 lux (light level 3) and 0.47 lux (light level 4). The illuminance at light level 4 was extrapolated from the other values, as the instrument was not sensitive enough to give reliable readings at this light level. Luminance of the test targets was measured with a Hagner photometer (Hagner AB, Solna, Sweden) by placing the sensor close to the stimulus monitor at the position of the target. During tests at a specific light level, a neutral density filter corresponding to the filter used on top of the decision box was placed in front of the monitor to match the decrease in illuminance. A table of light conditions at the four different light levels can be found in the electronic supplementary material, table S1.

Just inside the entrance to the decision box, next to the corridor, a set of infrared break beam sensors (Adafruit Industries, New York, USA) monitored the time when the bird entered. The behaviour of the bird in the decision box was monitored using a Pi NoIR camera module. The break beam sensor and camera module were controlled by single-board computers (Raspberry Pi 2, model B, Raspberry Pi, Pencoed, Wales).

### (c) Experiment 1: detection threshold

#### (i) Experimental procedures

**Figure 2.**
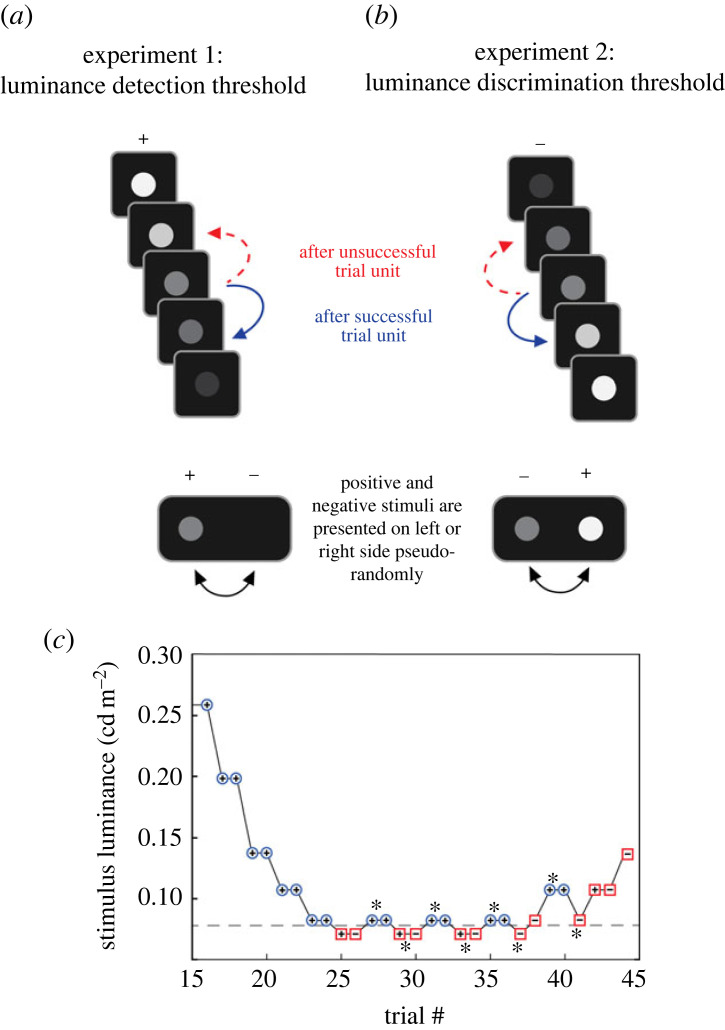
Example of stimuli from (*a*) experiment 1 and (*b*) experiment 2. The stimuli series (*a*,*b*) show stimuli of increasing difficulty from top to bottom. A successful trial unit (two consecutively correct trial choices including the same target luminance) resulted in an increase of difficulty (blue solid arrow), whereas an unsuccessful trial unit (either one incorrect trial choice or one correct choice followed by an incorrect trial choice including the same target luminance) resulted in a decrease in difficulty (red dashed arrow). (*c*) An example staircase from experiment 1. The plus and minus signs indicate trials with correct and incorrect choices, a blue coloured circle and red coloured squares indicate whether the choice belongs to a successful or unsuccessful trial unit. Asterisks indicate the reversals of the last 20 choices, which are included in the analysis of the threshold (dashed line). Note that the first reversal is removed from the analysis to give equal weight to successful and unsuccessful trial units.

We performed two experiments. Experiment 1 was designed to measure the detection threshold of the birds for a bright target on a dark background. At the start of each trial the bird was sitting on the waiting perch in the flight cage. In response to an auditory start signal, the bird flew or climbed up to the corridor and entered the decision box. As soon as the bird passed the break beam sensors, two stimuli were presented in the stimulus windows, one positive (rewarding) stimulus and one negative (unrewarding) stimulus. The negative stimulus was plain black while the positive stimulus was black with a bright circular target (figure 2*a*) of 2.2 cm diameter, and thus, extending 9.6° of the visual field of the bird when viewed from the decision point at the end of the dividing wall. The positive and negative stimuli were presented semi-randomly to the right and left according to Fellows [43]. The bird made a choice by approaching one of the stimuli. If the bird chose the positive stimulus, the feeder in front of it was opened and the bird was allowed to eat for a few seconds. If the bird chose the negative stimulus the feeder was not opened, and the target disappeared.

To initiate a new trial, the bird had to return to the waiting perch. The choice made by the bird was recorded manually and opening and closing of the feeder as well as the start of a new trial were controlled by the experimenter who watched the bird via the two cameras. The minimum luminance detection threshold was determined using a one-up/two-down staircase procedure [44]. If the bird made two consecutive correct choices on the same level of difficulty, this was considered as a successful trial unit, and the luminance of the positive stimulus was decreased in the next trial. By contrast, when the bird made one incorrect choice, or, alternatively, one correct choice followed by one incorrect choice, on the same difficulty level, this was considered an unsuccessful trial unit and the luminance was increased for the next trial (figure 2*a,c*). To increase the motivation of the birds in the beginning of a test session each staircase started at a degree well above the threshold of the birds. Step sizes were larger at the beginning of a staircase and were decreased at two occasions to be smallest near the threshold, for increased precision (electronic supplementary material, table S2). One staircase session included between 38 and 100 choices depending on how fast the bird reached a steady state performance, meaning that it did not improve its performance in further trials. Each bird participated in three staircase sessions in each of the four light levels.

To investigate whether the period of adaptation to the different light intensities in the experimental set-up had an influence on the test outcome we measured two different time intervals for each trial. We defined the response time as the period between the time points when a bird entered the decision box (registered by the infrared break beam sensor) and when it made a choice (manually registered by the experimenter). To estimate the time that a bird spent in the high light intensity in the flight cage we measured the inter-trial time as the period between the time when a bird left the decision box and the onset of a new trial.

#### (ii) Analysis of luminance detection threshold

The detection thresholds were analysed in absolute luminance values and not in Weber fractions, in relation to the background luminance, which would have been another possibility. The reason for this choice was that the background luminance at light levels 3 and 4 was below the absolute luminance threshold for budgerigars [34], why it would probably not influence the detection thresholds.

For every staircase session, the threshold was calculated as the average of the values at the reversals of the last 20 choices (figure 2*c*). To avoid estimation bias, we used an even number of reversals for each staircase session, excluding the first reversal if needed.

We used the r-package ‘lmerTest’ [45] in Rstudio (v. 1.4.1106 [46]) to fit linear mixed effect models (LMMs), by maximum likelihood, to the staircase detection thresholds (three samples for each bird and light level). The dependent variable (luminance detection threshold) was log-transformed to better fit the assumption of normality. To test whether the detection thresholds differed between the different light levels we compared a full model including light level as a fixed effect, to a reduced model excluding this fixed effect, with a likelihood ratio test. Individual bird was included in both models as a random intercept to avoid pseudo-replication. We used the ‘multcomp’ package [47] in RStudio to perform Tukey's *post hoc* test on the detection thresholds for the different light levels.

#### (iii) Analysis of time intervals

The response time and inter-trial time data are based on the same trials as the threshold data. The only exception are trials in which a bird entered and exited the decision box several times before making a choice. In these cases, the response time data were excluded from the analysis.

Response time data were fitted to an LMM. The dependent variable (response time) was inverse-transformed to better meet the assumption of normality. The full model included the main effects of inter-trial time, light level, successful/unsuccessful trial unit, as well as the interactions between inter-trial time and successful/unsuccessful trial unit and light level and successful/unsuccessful trial. Individual bird was included in all models as a random intercept to account for pseudo-replication. We used Akaike information criterion (AIC) to find the most likely model and a likelihood ratio test to compare models.

### (d) Experiment 2: discrimination threshold

#### (i) Experimental procedure

In experiment 2, we used the same test procedure as in experiment 1 to estimate the discrimination threshold for luminance differences between two simultaneously displayed targets. The positive stimulus consisted of a bright round target on a dark background with a constant luminance throughout the session (200, 10.4, 0.68 and 0.19 cd m^–2^ at levels 1 to 4, respectively). The negative stimulus was a target of lower luminance on the same dark background (figure 2*b*). Again, positive and negative stimuli were presented semi-randomly to the right and left according to Fellows [43]. The targets had the same size as the target in experiment 1 and were separated by 30.7° (centre to centre). Using the same one-up/two-down staircase procedure, and gradually decreased step sizes (electronic supplementary material, table S3), we tested the birds until the choices fluctuated around the discrimination threshold (figure 2*b*). The birds made between 35 and 100 choices in each staircase session. Two of the birds, bird 1 and bird 3, participated in three test sessions for each light level, whereas bird 2 participated in three sessions only for level 1, 2 and 3, and bird 4 participated in three sessions for level 1 and 2 and one session for level 3. As in experiment 1, we included the values at the reversals of the last 20 choices and used an even number of reversals.

#### (ii) Analysis of luminance difference threshold

The luminance difference thresholds are expressed in Weber fractions. According to Weber's law, the minimum detectable difference of a new stimulus is proportional to the value of the reference stimulus. In our experiment we calculate the Weber fraction *k* as

2.1 $$k=\frac{\Delta I}{I_{+}},$$

where Δ*I* is the luminance difference between the positive and the negative stimulus and *I_+_* is the luminance of the positive stimulus.

LMMs were fitted to the data and analysed in the same way as in experiment 1.

#### (iii) Analysis of time intervals

Response times and inter-trial times were measured and analysed in the same way as in experiment 1.

## 3. Results

### (a) Experiment 1

#### (i) Luminance detection threshold

**Figure 3.**
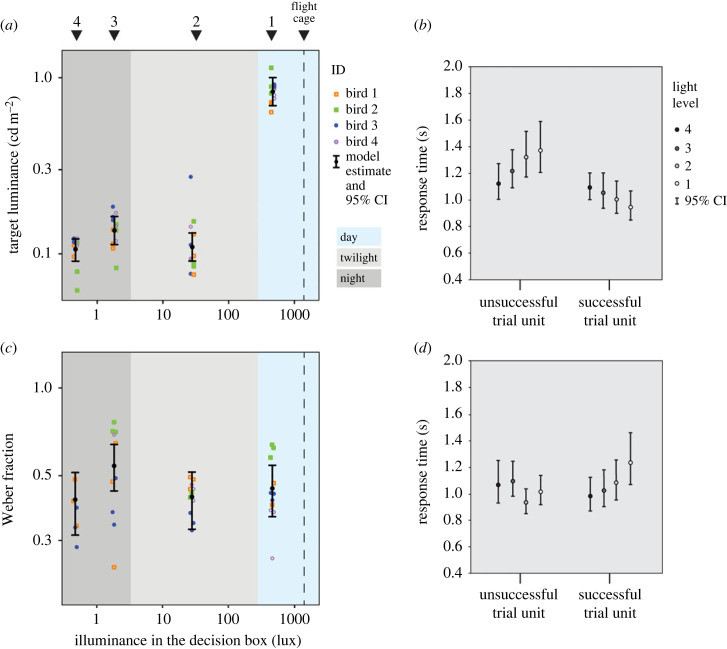
(*a,b*) Experiment 1: (*a*) detection threshold—the minimum luminance, at which birds could detect the target. Different colours and symbols represent results from individual birds, while black filled circles are the back-transformed model estimates of the thresholds. Numbers on top indicate illuminance in the decision box at the different light levels, dashed lines represent illuminance at the waiting perch in the flight cage. The definition of day, twilight and night illuminance follows Martin [1]. (*b*) Response times for trials in experiment 1 at each light level and separately for successful or unsuccessful trial units. Response times are the back-transformed estimates from the LMM. (*c,d*) Experiment 2: (*c*) discrimination threshold—the minimum luminance difference, expressed as Weber fraction (equation (2.1)), for which two bright targets could be discriminated. (*d*) Response times for experiment 2.

The results are based on a total of 48 staircase thresholds from four birds at four light levels. The birds were able to discriminate targets of similar luminance in the three darkest light levels (2–4), while the detection threshold on the brightest level (1) was considerably higher. Model comparison showed that a model including light level as a fixed effect and individual bird as a random effect had a significantly better fit to the data than a model only including individual bird as a random effect (*n* = 48, *χ*^2^_3_ = 126.7, *p* < 0.001). We therefore concluded that light level had an effect on detection threshold. At light levels 2 and 4 the estimated detection thresholds were similar (0.110 cd m^−2^, 95% confidence interval (CI) [0.092, 0.132], and 0.106 cd m^−2^, 95% CI [0.091, 0.123]) (figure 3*a*), while the threshold at level 3 was significantly higher (0.136 cd m^−2^, 95% CI [0.113, 0.163]) than at level 4 (*p* < 0.04). The threshold at level 1 (0.825 cd m^−2^, 95% CI [0.685, 0.988]) was higher than the thresholds at all other light levels (*p* < 0.001).

#### (ii) Time intervals

The median response time in experiment 1 was 1.17 s (*n* = 959 choices by four birds, first to third quartile = 0.93–1.60 s). The model that best explains the distribution of response times included the effect of light level (*F* = 3.27, *p* < 0.05), successful/unsuccessful trial unit (*F* = 24.14, *p* < 0.001), as well as the interaction between these two (*F* = 2.63, *p* < 0.05), and this model had a significantly better fit to the data compared to the null model (*χ*^2^_7_ = 35.9, *p* < 0.001). The birds had a shorter response time in successful trial units than in unsuccessful trial units. Median inter-trial time was 8.33 s (first to third quartile = 6.41–13.80 s).

### (b) Experiment 2

#### (i) Luminance discrimination threshold

The results include a total of 40 staircase thresholds from four individuals tested at four light levels. Light level had a significant effect on luminance discrimination threshold (*n* = 40, *χ*^2^_3_ = 10.07, *p* < 0.05). The smallest discriminable difference, a Weber fraction of 0.41 (95% CI [0.32, 0.53]) was found at level 4 (figure 3*c*). The threshold at level 1 was equivalent to a Weber fraction of 0.45 (95% CI [0.37, 0.55]), for level 2 it was 0.42 (95% CI [0.34, 0.53]), and for level 3 it was 0.54 (95% CI [0.46, 0.65]). The threshold at level 3 was significantly different to thresholds at level 2 (*p* < 0.05) and level 4 (*p* < 0.05).

#### (ii) Time intervals

The median response time in experiment 2 was 0.98 s (*n* = 799 choices by four birds, first to third quartile = 0.82–1.26 s). The most likely model included the fixed factors of inter-trial time (*F* = 11.7, *p* < 0.001), successful/unsuccessful trial unit (*F* = 32.5, *p* < 0.001) and light level (*F* = 11.2, *p* < 0.001), as well as the interaction between light level and successful/unsuccessful trial unit (*F* = 2.76, *p* < 0.05). This model had a significantly better fit to the data than the null model excluding all fixed effects (*χ*^2^_8_ = 95.0, *p* < 0.001). Median inter-trial time for experiment 2 was 7.79 s (first to third quartile = 6.17–11.13 s).

## 4. Discussion

By training budgerigars to leave a bright (1400 lux) flight cage, enter a dark decision box and detect or discriminate stimuli of different luminance, our experiment simulates the sudden drop in light intensity that cavity nesting birds experience when entering a dark nest to care for their offspring. In experiment 1 we investigated the luminance threshold for the detection of a bright target on a dark background and found that it depended on the light level in the decision box. The detection threshold for the lower three light levels (0.47–28 lux) were in the same range, between 0.106 and 0.136 cd m^−2^, while the threshold for the brightest level (469 lux) was considerably higher, 0.825 cd m^−2^ (figure 3*a*). We assume that the luminance of the background at the dimmest levels was too dim to be detected by the bird, hence the similar results for these conditions. The lower luminance detection thresholds in the dimmer light levels suggest that the birds have a fast mechanism allowing them to adapt to these low levels.

In experiment 2, we determined the luminance discrimination thresholds for two bright targets on a dark background. The thresholds were in the same range at all four different light levels, with Weber fractions between 0.41 (level 4) and 0.54 (level 3; figure 3*c*). Without any adaptation we would have expected a gradual rise in Weber fraction with decreasing illuminance in the decision box [48]. In our data no such trend could be seen, indicating the existence of a fast luminance adaptation mechanism.

We cannot determine the mechanisms underlying the high sensitivity observed in the dim light conditions, but we can conclude that they function within about 1 s. As mentioned in the introduction, pupil dynamics probably play a minor role in this context [3,4]. Only few birds are capable of remarkable changes in pupil size. The king penguin (*Aptenodytes patagonicus)* constricts its pupils to tiny square-shaped pinholes in daylight allowing the retina to stay dark-adapted before diving to foraging grounds several hundred metres below the sea surface [49]. Dilation of the pupils to its maximum size under water increases retinal image illumination 300-fold. No such extreme pupil dynamics have been reported in any terrestrial bird species. Budgerigars are able to dilate their pupils from 2.3 mm to 3 mm, allowing their eyes to let in 1.7 times more light [4]. This can only account for a small fraction of the sensitivity increase seen between levels 1 and 2, in our experiments. Avian pupil constriction can happen within the tenth of a second, an ability attributed to the presence of striated muscle [3,50,51]. Pupil dilation, by contrast, requires several seconds to be completed [51,52]. The birds in our experiments took only around 1 s to locate the correct stimuli, clearly excluding pupil dynamics as the main mechanism of sensitivity increase.

In birds, fast visual adaptation has only been studied in the context of colour constancy. A study on chickens [53] indicates the presence of fast (or simultaneous) and slower (up to 5 min) adaptational mechanisms in the chromatic pathway. Primates have fast mechanisms which adjust retinal luminance sensitivity within less than a second [20,54], enabling retinal adaptation to the highly dynamic luminance variation experienced when actively exploring a visual scene [18,55]. Birds have a similar rate of gaze change as humans when scanning the environment [56] and thus probably need similarly rapid luminance adaptation.

We are not sure why, in both experiments, lower thresholds were found at light levels 2 and 4 than at the intermediate level 3 (figure 3*a,c*). Previous experiments have indicated that the single cones of budgerigars loose sensitivity at an illuminance between 2 and 10 lux, but double cones remain active at lower intensities ([7], and O. Lind 2013, unpublished data). This shift, happening between light levels 2 and 3 might explain at least part of the sensitivity drop seen at level 3.

In a previous study on brightness discrimination, budgerigars had a Weber fraction of 0.18 when tested with two spatially separated large achromatic fields [35]. In our measurements, we found considerably higher thresholds, a Weber fraction between 0.41 and 0.54, a difference probably resulting from the short adaptation period.

The median response time of the budgerigars was 1.17 s in experiment 1 and 0.98 s in experiment 2. Whether the choice was part of a successful or unsuccessful trial unit, had the largest effect on response time. Our expectation had been that longer response times would reflect longer adaptation periods, and thus, correlate with successful trials. In both experiments, birds had a shorter response time in successful trials, but the differences were too small (within the range of 10^−2^ s) to allow conclusions about effects on adaptation. Shorter response times for correct than for incorrect choices have previously been found in studies of optimal decision making [57,58] and thus, are more likely a consequence of decision making than of adaptation. In primates the initial steep sensitivity rise (within 1 s) is followed by a slower adaptive change [9]. If the time course of early adaptation is similar in birds, then fast decision taking is probably an efficient behaviour, as feeding parents commute to the nest many times every day, and long decision times will come with a high cost [57]. The fast adaptation mechanisms are incomplete but sufficient to allow birds to see well enough in the nest.

Unlike many passerine chicks, budgerigar chicks do not beg for food with a wide-open gape with conspicuous flanges. Budgerigars are unable to lift the head until they are 6–8 days old and although they are able to vocalize, active begging behaviour (e.g. head-bobbing, moving towards the parent) are uncommon until they are around 11 days old [59,60]. During this period the parents initiate feeding events by beak-grasping [59]. Like other psittacine birds, budgerigars have specialized touch-receptors, referred to as the ‘bill-tip organ’, in the upper bill [61,62]. The bill-tip organ is used in object exploration and manipulation [61,62] and it is possible that budgerigars use tactile stimuli to a larger extent than visual stimuli in parent-offspring communication. Nevertheless, budgerigar chicks are individually targeted during feeding suggesting visual detection is involved [59]. Furthermore, the eggs and chicks of a domesticated budgerigar nest have a Weber contrast of about 0.4–0.6 to a nest background made of wood chips (electronic supplementary material, figure S1 and table S8). The typical substrate on which wild budgerigars lay their eggs consists of decaying wood and faeces [63] which probably provides an even higher contrast. Our study suggests that budgerigars adapt to the strong drop in light intensity, equivalent to that experienced when entering the nest, within less than 1 s. Their sensitivity then allows them to fast and efficiently feed the chicks using visual control.

## Data Availability

All data used in this manuscript are available from the Dryad Digital Repository: https://doi.org/10.5061/dryad.qz612jmm8 [64]. The data are also provided in the electronic supplementary material [65].
